# Integrated transcriptomics identifies immune–metabolic dysregulation and candidate diagnostic biomarkers in oligoasthenozoospermia with experimental validation

**DOI:** 10.3389/fcell.2026.1832331

**Published:** 2026-06-05

**Authors:** Lei Peng, Jie Su, Zhi Zhang, Yikang Yu, Ziyan Chen, Guiyuan Lv, Suhong Chen

**Affiliations:** School of Pharmaceutical Sciences, Zhejiang Chinese Medical University, Hangzhou, Zhejiang, China

**Keywords:** diagnostic biomarkers, immune-related signatures, machine learning, oligoasthenozoospermia, spermatogenesis, WGCNA

## Abstract

**Objective:**

Oligoasthenozoospermia is a major cause of male infertility and is characterized by reduced sperm concentration and motility. However, candidate molecular biomarkers and integrated mechanistic frameworks for disease characterization remain limited. This study aimed to identify candidate diagnostic biomarkers for oligoasthenozoospermia and to characterize the immune–metabolic dysregulation associated with impaired spermatogenesis.

**Methods:**

This study integrated gene expression profiles from two Gene Expression Omnibus (GEO) datasets, GSE45887 and GSE45885, to analyze transcriptomic alterations in oligoasthenozoospermia. After data normalization and batch correction, differential expression analysis and weighted gene co-expression network analysis (WGCNA) were performed to identify disease-related genes. Functional enrichment was further evaluated using Gene Set Variation Analysis (GSVA) and Gene Set Enrichment Analysis (GSEA). Immune-related transcriptomic signature variation was estimated by single-sample gene set enrichment analysis (ssGSEA). To identify candidate biomarkers, three machine learning algorithms—least absolute shrinkage and selection operator (LASSO), random forest, and support vector machine-recursive feature elimination (SVM-RFE)—were applied to screen core genes, followed by construction and internal evaluation of logistic regression, LASSO, random forest, and support vector machine (SVM) diagnostic models. Finally, a smoking- and ethanol-induced mouse model of oligoasthenozoospermia was established, and sperm quality, histopathology, transcriptomic alterations, immunofluorescence, and Western blotting were used for experimental validation.

**Results:**

Differential expression analysis identified reproducible transcriptomic alterations in oligoasthenozoospermia, and intersection with WGCNA-derived key module genes yielded 86 candidate genes. Functional enrichment analysis showed that these genes were mainly associated with immune- and metabolism-related pathways. GSVA and GSEA demonstrated coordinated activation of complement, IL6-JAK-STAT3, interferon-γ, and PI3K-AKT-mTOR/mTORC1 signaling, together with marked suppression of spermatogenesis-related programs. Immune signature analysis based on ssGSEA suggested potential immune microenvironment alterations at the transcriptomic level and correlations between key genes and multiple immune-related signatures. Further analysis using three machine learning algorithms identified 12 core genes. Among the tested classifiers, the random forest model showed the best overall performance in internal validation; however, the near-perfect performance observed in several models should be interpreted cautiously given the limited sample size. In the mouse model, sperm concentration, viability, and motility were significantly decreased, whereas the sperm abnormality rate was significantly increased, accompanied by abnormal testicular histology and reduced PCNA expression. Transcriptomic and protein-level validation further supported dysregulation of representative candidate genes and pathways, supporting the biological relevance of the human transcriptomic findings rather than providing exhaustive mechanistic validation.

**Conclusion:**

This study identified a 12-gene candidate biomarker panel for oligoasthenozoospermia and revealed a coordinated immune–metabolic–spermatogenic dysregulation pattern. These findings provide a transcriptomic framework for molecular characterization of oligoasthenozoospermia and preliminary evidence for future biomarker-based diagnostic development, which requires external validation in larger independent and more clinically homogeneous human cohorts.

## Introduction

Oligoasthenozoospermia represents one of the most prevalent clinical phenotypes of male infertility and is directly associated with impaired natural conception and the selection of assisted reproductive strategies (Kuribayashi et al., 2026). Current clinical evaluation primarily relies on conventional semen analysis, including sperm concentration, motility, and morphology. Although these parameters are indispensable for disease grading and therapeutic monitoring, they reflect terminal phenotypes rather than underlying molecular drivers and provide limited mechanistic insight or predictive value for individualized management (Hongyu et al., 2025). Furthermore, semen parameters are highly influenced by inter-individual variability, environmental exposures, and sampling fluctuations, resulting in substantial biological heterogeneity within the same diagnostic category (Lin et al., 2024). Consequently, the absence of reliable molecular biomarkers hampers early risk stratification and precision intervention in oligoasthenozoospermia.

In recent years, transcriptomic, proteomic, and metabolomic profiling studies have sought to uncover dysregulated molecular pathways in male infertility (Hongyu et al., 2025; Yan et al., 2025; Li et al., 2022). These investigations have implicated immune activation, oxidative stress, metabolic disturbance, and spermatogenic dysfunction; however, reproducibility across independent cohorts remains limited. Candidate diagnostic biomarkers with cross-cohort robustness and experimental validation remain insufficiently established. Moreover, many predictive models derived from omics data are constructed without biological confirmation, thereby restricting their translational applicability. Thus, there is an urgent need to establish an integrated and experimentally validated biomarker framework that connects immune microenvironment remodeling, metabolic signaling perturbation, and spermatogenic impairment.

The testis is a classical immune-privileged organ in which normal spermatogenesis depends on the integrity of the blood–testis barrier and a finely regulated local immune tolerance network (Zhong et al., 2026). Disruption of immune homeostasis—triggered by genetic susceptibility, environmental exposure, or lifestyle-related stress—may result in persistent low-grade inflammation, impairing Sertoli–germ cell communication and microenvironmental stability (Batool, 2025; Bao et al., 2026). Concurrently, spermatogenesis is an energy-intensive process requiring tightly controlled lipid metabolism, protein synthesis, and growth signaling. The mTOR and PI3K–AKT pathways, among others, play central roles in maintaining cellular homeostasis, autophagy balance, and differentiation dynamics (Zhao et al., 2024; Yang et al., 2025). Importantly, extensive crosstalk exists between inflammatory signaling and metabolic regulation; inflammatory stimuli can induce metabolic reprogramming, while metabolic disturbance can further amplify inflammatory responses, forming a maladaptive feedback loop (Cai et al., 2022). Emerging evidence suggests that such immune–metabolic disequilibrium compromises spermatogonial stem cell maintenance, meiotic progression, and sperm maturation quality, constituting a key pathological basis of male infertility (Hed and ger, 2016; Cai et al., 2022). However, a systematic characterization of this coordinated dysregulation axis in oligoasthenozoospermia remains incomplete.

Despite the widespread application of multi-omics technologies and bioinformatics approaches in reproductive medicine, several limitations persist (Panner Selvam et al., 2020; Liu et al., 2024). Many prior studies rely on single cohorts with modest sample sizes, rendering findings susceptible to cohort-specific bias. Mechanistic analyses often focus on isolated genes or pathways, lacking systems-level integration of immune regulation, metabolic signaling, and spermatogenic programs. Furthermore, immune cell infiltration patterns and their coupling with reproductive-related gene expression have not been comprehensively delineated in oligoasthenozoospermia. Although machine learning–based predictive models have been proposed, feature selection strategies are frequently heterogeneous, and independent biological validation is seldom performed (Dias et al., 2019). The absence of an integrated “computational discovery–experimental validation” pipeline limits confidence in causal relevance and clinical translation.

However, publicly available human testicular transcriptomic datasets for oligoasthenozoospermia remain extremely limited, and the available cohorts may exhibit incomplete clinical annotation and a degree of phenotypic heterogeneity. Therefore, integrative analyses based on currently accessible public resources should be interpreted as exploratory efforts to identify candidate molecular alterations and biomarker signals rather than definitive disease-specific molecular signatures.

**FIGURE 1:**
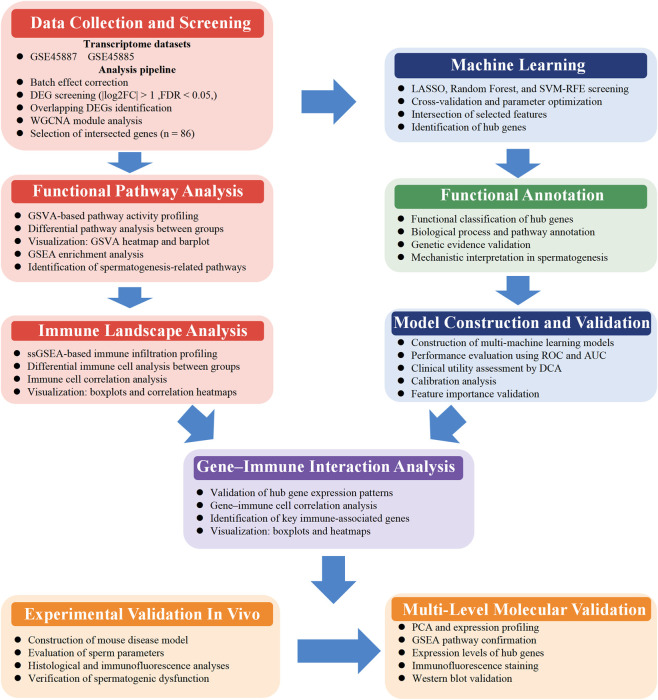
Overall workflow of the integrative bioinformatics, machine learning, and experimental validation framework for oligoasthenozoospermia.

In the present study, we integrated multi-cohort transcriptomic datasets with bioinformatic mining and ensemble machine learning strategies to identify candidate diagnostic biomarkers of oligoasthenozoospermia. We further performed immune-related transcriptomic signature profiling and pathway activity analysis to delineate a coordinated immune–metabolic–spermatogenic dysregulation axis. Key candidate biomarkers were validated in a smoking- and ethanol-induced mouse model, establishing concordance between computational predictions and biological phenotypes (workflow shown in Figure 1). Through this integrative approach, we aim to define candidate molecular biomarkers and construct an interpretable diagnostic model that may support molecular stratification, early risk assessment, and biomarker-driven precision intervention in male infertility.

## Materials and methods

### Data processing

Gene expression profiles for oligoasthenozoospermia were retrieved from the Gene Expression Omnibus (GEO), including GSE45887 and GSE45885, both derived from human testicular tissue. GSE45887 comprised 4 normal controls and 16 oligoasthenozoospermia samples (n = 20), whereas GSE45885 included 4 normal controls and 27 oligoasthenozoospermia samples (n = 31). Probe-level data were reannotated to official gene symbols according to platform files, and expression values of multiple probes mapping to the same gene were averaged. Data preprocessing was performed using the limma package in R, including background correction, quantile normalization, and log_2_ transformation. Batch effects between datasets were corrected using the ComBat function in the sva package. Boxplots were generated after correction to assess distribution consistency across samples prior to integrative analysis. Because publicly available human testicular transcriptomic datasets specifically focused on oligoasthenozoospermia are extremely limited, especially those with well-matched normal controls, the present study integrated the few analyzable datasets currently accessible in GEO. Accordingly, the number of control samples available for integrative analysis was small, which was taken into consideration during data interpretation.

### Identification of differentially expressed genes

Differential expression analysis between oligoasthenozoospermia and control samples was performed using the *limma* package in R. A linear model was fitted to the normalized expression matrix, and empirical Bayes moderation was applied to adjust the standard errors. Genes meeting the criteria of |log2 fold change (log2FC)| > 1 and false discovery rate (FDR) < 0.05 were defined as differentially expressed genes (DEGs). The distribution of DEGs was visualized using a volcano plot.

### Weighted gene co-expression network analysis (WGCNA)

Weighted gene co-expression network analysis was performed using the WGCNA package in R to identify disease-associated gene modules. The top 5,000 genes ranked by median absolute deviation were selected for network construction. The goodSamplesGenes function was first used to assess sample and gene quality. Hierarchical clustering of samples was then performed to detect potential outliers, and no obvious outlier samples requiring removal were identified. Candidate soft-thresholding powers were evaluated using the pickSoftThreshold function according to the scale-free topology fit index and mean connectivity, and a soft-thresholding power (β) of 10 was selected for network construction. An adjacency matrix was subsequently constructed and transformed into a topological overlap matrix (TOM). Genes were hierarchically clustered based on TOM dissimilarity, and modules were identified using the dynamic tree cut algorithm with minModuleSize = 20, deepSplit = 2, and pamRespectsDendro = FALSE. Similar modules were then merged at a cut height of 0.25 based on module eigengene similarity, and module eigengenes (MEs) were calculated. Correlation analysis between MEs and clinical traits was performed to identify key modules associated with oligoasthenozoospermia. Module membership and gene significance were further evaluated for downstream interpretation.

### Gene set variation analysis and enrichment validation (GSVA/GSEA)

Gene set variation analysis (GSVA) was performed using the GSVA R package to evaluate pathway activity across samples based on the normalized expression matrix. Hallmark gene sets (MSigDB v2025.1) were used as reference, and enrichment scores were calculated with default parameters. Differential pathway activity between the oligoasthenozoospermia and control groups was assessed using the *limma* package based on the GSVA score matrix, and results were visualized accordingly. To further validate the robustness and directionality of key pathways identified by GSVA, gene set enrichment analysis (GSEA) was conducted using the *clusterProfiler* package. Enrichment significance was determined according to normalized enrichment score (NES), nominal *P* < 0.05, and false discovery rate (FDR) < 0.25.

### Immune-related transcriptomic signature analysis and correlation assessment

Immune-related transcriptomic signature variation was estimated using single-sample gene set enrichment analysis (ssGSEA) implemented in the GSVA R package. Immune cell–related marker genes were extracted from a predefined immune signature file and organized into gene sets according to cell type. Based on the normalized expression matrix, ssGSEA scores were calculated for each sample using default parameters, generating a signature score matrix representing the relative enrichment of immune-related signatures. Differences in immune-related signatures between the oligoasthenozoospermia and control groups were evaluated using the Wilcoxon rank-sum test, and statistical significance was visualized with boxplots generated by the ggpubr package. To further explore relationships among immune-related signatures, Spearman correlation analysis was performed on the ssGSEA score matrix within the disease group.

### Multi-algorithm feature selection and importance evaluation

To identify candidate diagnostic biomarkers, multiple machine learning algorithms were applied for feature selection. First, Least Absolute Shrinkage and Selection Operator (LASSO) regression was performed using a generalized linear model based on the gene expression matrix of disease and control samples. Ten-fold cross-validation was used to determine the optimal penalty parameter (λ), and genes with non-zero regression coefficients at the minimum cross-validation error were retained as candidate feature genes. Subsequently, a random forest classifier was constructed to evaluate feature importance. The contribution of each gene to classification performance was quantified using the mean decrease in Gini index, and genes were ranked according to their importance scores. Model stability was assessed by examining the out-of-bag error rate across different tree numbers. Finally, Support Vector Machine–Recursive Feature Elimination (SVM-RFE) was applied to further refine the candidate genes. Features were iteratively removed based on their weights in the SVM model, and cross-validation was used to determine the optimal number of features by minimizing classification error. Genes consistently identified across these algorithms were considered key feature genes for subsequent modeling and validation.

### Machine learning model construction and internal validation

Based on the selected feature genes, all samples were randomly divided into a training set and a testing set at a ratio of 7:3. Logistic regression, LASSO regression, random forest, and SVM classifiers were constructed, with model training and parameter tuning performed in the training cohort. Model performance was evaluated in the testing cohort using receiver operating characteristic (ROC) curves and the area under the curve (AUC) to assess discriminative ability. Additional metrics, including accuracy, sensitivity, specificity, precision, F1 score, and Brier score, were calculated for comprehensive comparison. Decision curve analysis (DCA) was conducted to estimate the clinical net benefit of each model across a range of threshold probabilities. Calibration analysis was further performed for the optimal model to evaluate the agreement between predicted and observed probabilities. Because no sufficiently appropriate independent external human public cohort was available, model performance was assessed by internal validation only. Accordingly, the reported AUC values reflect internal model evaluation within the current dataset rather than external generalizability.

### Functional annotation and integration of genetic evidence for candidate genes

Publicly available bioinformatics databases were used to perform systematic functional annotation and genetic evidence integration for the selected candidate genes. Gene full names, primary molecular functions, and key biological processes were summarized, including roles in transcriptional regulation, signal transduction, metabolic regulation, and cellular structural maintenance. Genetic regulatory evidence was further integrated, including expression quantitative trait loci (eQTL), splicing quantitative trait loci (sQTL), and protein quantitative trait loci (pQTL), to evaluate tissue-specific regulatory patterns and potential associations with disease-related phenotypes. By integrating functional annotation, genetic evidence, and published literature, the potential biological roles of candidate genes in spermatogenesis were comprehensively assessed, providing a theoretical basis for subsequent mechanistic investigations.

### Establishment of the animal model

Twelve male ICR mice (SPF grade, 6–8 weeks old, 25–30 g) were obtained from Hangzhou Qizhen Laboratory Animal Co., Ltd (License No. SCXK (Zhe) 2022–0005). All animals were housed at the Experimental Animal Center of Zhejiang Chinese Medical University under standard conditions (22 °C ± 2 °C, 50% ± 10% humidity, 12 h light/dark cycle) with free access to food and water. After 1 week of acclimatization, mice were randomly assigned to the normal control group and the model group. The control group received no intervention. The oligoasthenozoospermia model was established in the model group using combined cigarette smoke exposure and ethanol administration. Specifically, mice were exposed to cigarette smoke every other day for 30 min per session, while simultaneously receiving 10% (v/v) ethanol in drinking water *ad libitum* for 4 consecutive months. All animal experiments were approved by the Animal Care and Use Committee of Zhejiang Chinese Medical University (Approval No. 20240108–08) and were conducted in accordance with institutional and national guidelines for laboratory animal care.

### Sperm quality assessment

At the end of the 4-month intervention, mice were deeply anesthetized with isoflurane (3%–5% in oxygen) and euthanized by cervical dislocation. Death was confirmed by cessation of respiration and heartbeat before immediate collection of testicular and epididymal tissues. Under sterile conditions, the epididymides were gently minced to release spermatozoa. The tissue fragments were removed, and the suspension was incubated at 37 °C for 5 min to obtain a sperm suspension. For sperm concentration measurement, 10 μL of the suspension was loaded onto a hemocytometer and examined under a light microscope. Sperm density was determined using the standard counting method. Sperm motility was evaluated by randomly assessing 200 spermatozoa and classifying them into four grades: grade I (rapid progressive motility), grade II (slow progressive or curvilinear motility), grade III (non-progressive motility), and grade IV (immotile). Sperm motility (%) was calculated as (grade I + II + III sperm)/200% × 100%. For sperm viability analysis, 10 μL of sperm suspension was mixed with an equal volume of 0.15% eosin solution, smeared onto a slide, and 500 spermatozoa were randomly counted. Viability was determined based on head staining. For sperm morphology assessment, 80 μL of sperm suspension was mixed with 20 μL of 1% eosin solution and incubated for 15 min before smear preparation. A total of 500 intact spermatozoa were examined under a microscope, and the percentage of morphologically abnormal sperm was calculated.

### Histopathological analysis

Testicular tissues were harvested and fixed in 4% paraformaldehyde solution, followed by routine dehydration and paraffin embedding. Serial sections (4 μm thickness) were prepared. After deparaffinization and rehydration, sections were stained with hematoxylin and eosin (H&E). Histological morphology and pathological alterations of the testes were examined and imaged under a light microscope.

### RNA sequencing and transcriptomic analysis

Total RNA was isolated from mouse testicular tissues and subjected to quality assessment prior to library preparation. Libraries were constructed from qualified RNA samples and sequenced on the NovaSeq X Plus platform. After quality control, alignment, and expression quantification, a gene expression matrix was obtained for downstream analysis. GSEA was performed to examine the enrichment of representative pathways identified in the human transcriptomic analysis. The expression patterns of selected candidate genes were also evaluated in the mouse model.

### Immunofluorescence staining

Paraffin-embedded mouse testicular sections (4 μm) were deparaffinized, rehydrated, and subjected to antigen retrieval. After blocking to reduce nonspecific binding, the sections were incubated overnight at 4 °C with primary antibodies against PCNA (Proteintech, 10205-2-AP), PILRB (HuaBio, et1608-64), IL1RN (Abbkine, A21089), FER1L5 (Abbkine, 014660101), and GLT6D1 (Bioss, BF01305394). After washing with PBS, the sections were incubated with fluorophore-conjugated secondary antibodies at room temperature in the dark. Nuclei were counterstained with DAPI. Images were captured using a fluorescence microscope (Zeiss AXIOSCOPE. A1, Germany).

### Western blot analysis

Total protein was extracted from mouse testicular tissues using RIPA lysis buffer containing PMSF. Protein concentrations were measured using a BCA protein assay kit. Equal amounts of protein were separated by SDS-PAGE, transferred onto PVDF membranes, and blocked with 5% non-fat milk. The membranes were then incubated overnight at 4 °C with primary antibodies against PILRB (HuaBio, et1608-64), IL1RN (Abbkine, A21089), and CATSPER4 (HuaBio, ha601101), followed by incubation with HRP-conjugated secondary antibodies for 2 h at room temperature. Protein signals were detected using enhanced chemiluminescence reagents and quantified with ImageJ software.

### Statistical analysis

Statistical analyses of *in vivo* experimental data were performed using IBM SPSS Statistics 22.0. Normality was assessed using the Shapiro–Wilk test. For comparisons between two groups (normal group vs. model group), normally distributed data are presented as mean ± SD and were analyzed using an unpaired two-tailed Student’s t test; non-normally distributed data are presented as median (interquartile range) and were analyzed using the Mann–Whitney U test. All tests were two-sided, and *P* < 0.05 was considered statistically significant.

## Results

### Identification of differentially expressed genes and construction of weighted gene co-expression network

**FIGURE 2:**
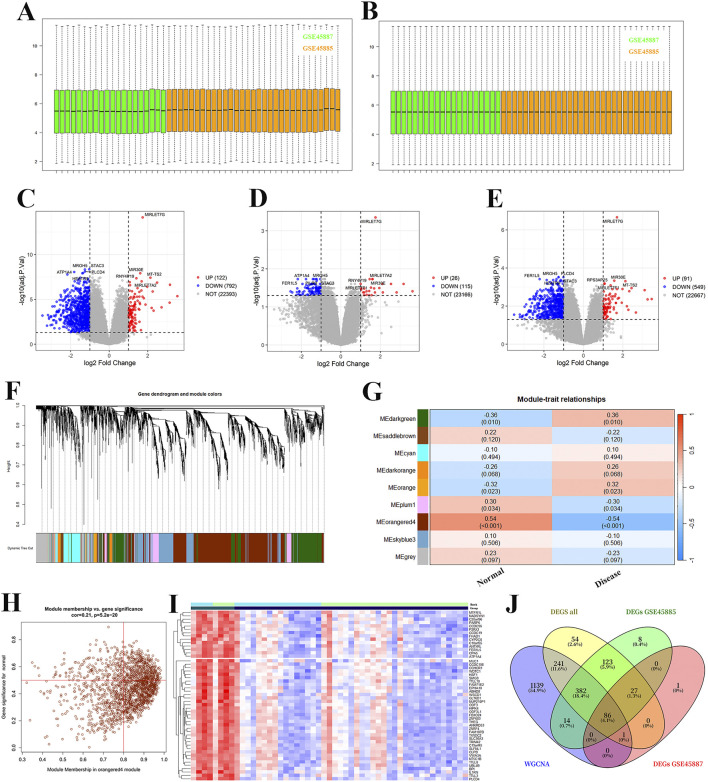
Identification of differentially expressed genes and key co-expression modules associated with oligoasthenozoospermia. **(A,B)** Boxplots showing the distribution of gene expression levels in GSE45887 and GSE45885 before and after normalization. **(C)** Volcano plot of differentially expressed genes (DEGs) in the merged dataset. **(D)** Volcano plot of DEGs in the GSE45887 dataset. **(E)** Volcano plot of DEGs in the GSE45885 dataset. **(F)** Gene clustering dendrogram and module assignment based on weighted gene co-expression network analysis (WGCNA). **(G)** Heatmap of module–trait relationships between gene modules and clinical phenotypes **(H)** Scatter plot showing the correlation between module membership and gene significance in the key module. **(I)** Heatmap of expression patterns of genes in the key module between normal and disease groups. **(J)** Venn diagram showing the overlap among DEGs from different datasets and WGCNA-identified module genes.

To systematically characterize gene expression alterations associated with oligoasthenozoospermia, the GSE45887 and GSE45885 datasets were first normalized and batch-corrected. Boxplot analysis demonstrated that, after preprocessing, the distribution of gene expression values across samples became comparable, indicating satisfactory data quality for downstream analyses (Figures 2A,B).

Based on the merged dataset, differential expression analysis identified 122 upregulated genes and 792 downregulated genes in the disease group compared with controls (Figure 2C). Independent analyses of the two datasets showed consistent trends: in GSE45887, 26 upregulated and 115 downregulated genes were identified (Figure 2D), while in GSE45885, 91 upregulated and 549 downregulated genes were detected (Figure 2E). These findings indicate directionally consistent differential expression patterns across the available cohorts, supporting subsequent integrative analyses while acknowledging the limitations of public sample size and clinical heterogeneity.

A weighted gene co-expression network was then constructed using the top 5,000 genes ranked by median absolute deviation. Sample clustering did not identify any obvious outlier samples requiring exclusion. Based on scale-free topology analysis, a soft-thresholding power of 10 was selected for network construction. Genes were subsequently grouped into multiple color-coded modules after dynamic tree cutting and module merging (Figure 2F). Module–trait relationship analysis revealed that several modules were significantly associated with disease status (Figure 2G). Notably, the orangered4 module (MEorangered4) showed a significant negative correlation with oligoasthenozoospermia (r = −0.54, *P* < 0.001) and a positive correlation with normal phenotype, suggesting its potential protective or downregulated role in disease progression.

Further analysis demonstrated a significant positive correlation between module membership and gene significance within the orangered4 module (Figure 2H), indicating that genes in this module were highly concordant with disease phenotype. Hierarchical clustering of genes in the key module showed clear separation between disease and control samples at the expression level (Figure 2I).

Intersection analysis between differentially expressed genes and WGCNA module genes identified 86 overlapping genes (Figure 2J), which were retained as candidate genes for subsequent feature selection and exploratory mechanistic investigation.

### Gene set enrichment analysis

**FIGURE 3:**
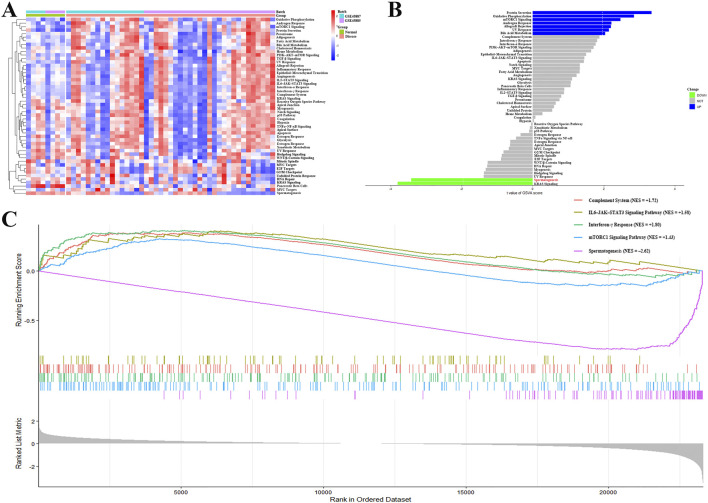
Pathway activity profiling and enrichment analysis based on GSVA and GSEA. **(A)** Heatmap showing GSVA scores of Hallmark pathways in normal and oligoasthenozoospermia samples. **(B)** Bar plot displaying differentially activated pathways based on GSVA score comparison between groups. **(C)** Gene set enrichment analysis (GSEA) plots of representative pathways, including complement system, IL6-JAK-STAT3 signaling, interferon response, mTORC1 signaling, and spermatogenesis.

To characterize pathway-level alterations in oligoasthenozoospermia, Gene Set Variation Analysis (GSVA) was performed based on the normalized expression matrix. The GSVA heatmap demonstrated distinct pathway activity patterns between disease and control samples, with a clear global separation in pathway activity profiles (Figure 3A), indicating widespread biological dysregulation in affected individuals.

Differential analysis of GSVA scores demonstrated that multiple pathways were altered in the disease group (Figure 3B). The complement signaling pathway, IL6–JAK–STAT3 signaling pathway, interferon-γ signaling pathway, and mTORC1/PI3K–AKT–mTOR signaling pathway exhibited an overall upward trend, whereas the spermatogenesis-related pathway was significantly downregulated. These results suggest a tendency toward activation of immune-inflammatory and metabolic signaling accompanied by a statistically significant suppression of spermatogenic programs in oligoasthenozoospermia.

Based on the GSEA results (Figure 3C), the complement system (NES = +1.72), IL6–JAK–STAT3 signaling pathway (NES = +1.58), interferon-γ response (NES = +1.80), and mTORC1 signaling pathway (NES = +1.43) exhibited positive normalized enrichment scores, indicating enrichment trends in the disease group. In contrast, the spermatogenesis pathway demonstrated a strongly negative normalized enrichment score (NES = −2.62), indicating significant enrichment in the control group and marked suppression in oligoasthenozoospermia samples. These findings suggest that immune-inflammatory and metabolic signaling pathways display coordinated enrichment tendencies in the disease condition, whereas spermatogenesis-related programs are significantly downregulated. The pronounced negative enrichment of the spermatogenesis pathway supports impaired spermatogenic activity as a central molecular feature of oligoasthenozoospermia, while immune and metabolic pathways show directionally consistent activation patterns.

### Transcriptome-inferred immune signature patterns and correlation analysis

**FIGURE 4:**
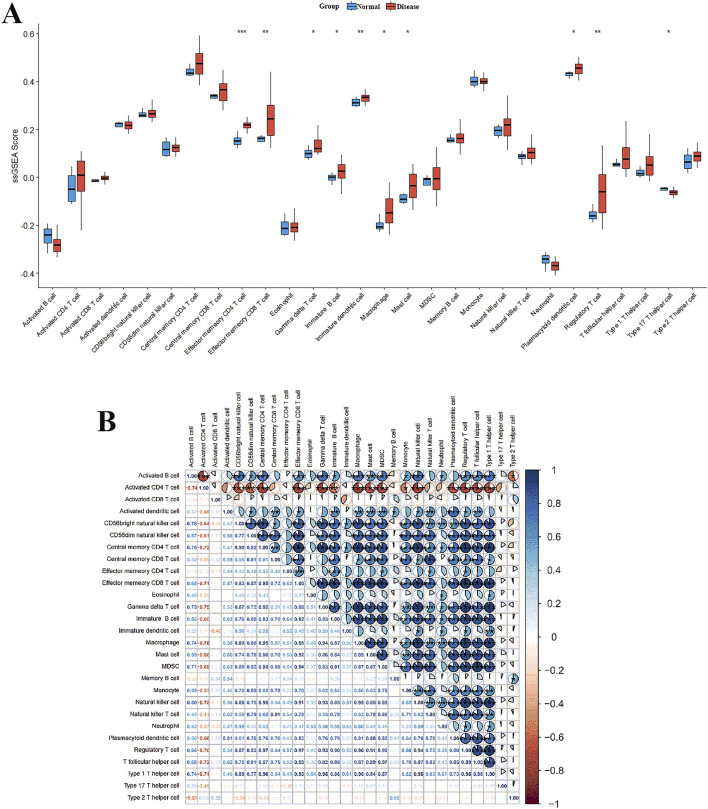
Transcriptome-inferred immune signature patterns and correlation analysis based on ssGSEA .**(A)** Comparison of ssGSEA scores of immune-related signatures between normal and oligoasthenozoospermia groups. Statistical significance was evaluated using the Wilcoxon rank-sum test (*P < 0.05, **P < 0.01, ***P < 0.001). **(B)** Spearman correlation analysis of ssGSEA-derived immune-related signatures in disease samples. Circle size and color represent the strength and direction of correlations.

Immune-related transcriptomic signature variation was estimated using the ssGSEA algorithm, and differences between the oligoasthenozoospermia and control groups were compared. Significant differences in multiple immune-related signatures were observed between the two groups (Figure 4A). Compared with controls, the disease group exhibited significant differences in signatures corresponding to activated B Cells, CD8^+^ T Cells, effector memory T Cells, and natural killer cells (*P* < 0.05), suggesting potential immune microenvironment alterations at the transcriptomic level in oligoasthenozoospermia.

Correlation analysis was subsequently performed within the disease group to evaluate relationships among immune-related signatures. Spearman correlation analysis revealed significant positive and negative correlations among different ssGSEA-derived immune signatures (Figure 4B). T cell–related signatures showed strong positive correlations with each other, whereas certain innate immune-related signatures showed inverse associations with adaptive immune-related signatures, suggesting coordinated yet heterogeneous transcriptome-level immune variation. Collectively, these findings suggest that oligoasthenozoospermia may be associated with transcriptome-inferred immune microenvironment alterations and changes in the relationships among immune-related signatures. However, these observations are based on bulk transcriptomic inference and should not be interpreted as direct evidence of immune cell composition changes without further marker-based validation.

### Feature gene selection and machine learning model construction

To further identify diagnostically relevant genes, multiple machine learning algorithms, including LASSO regression, random forest, and support vector machine–recursive feature elimination (SVM-RFE), were applied for feature selection.

**FIGURE 5:**
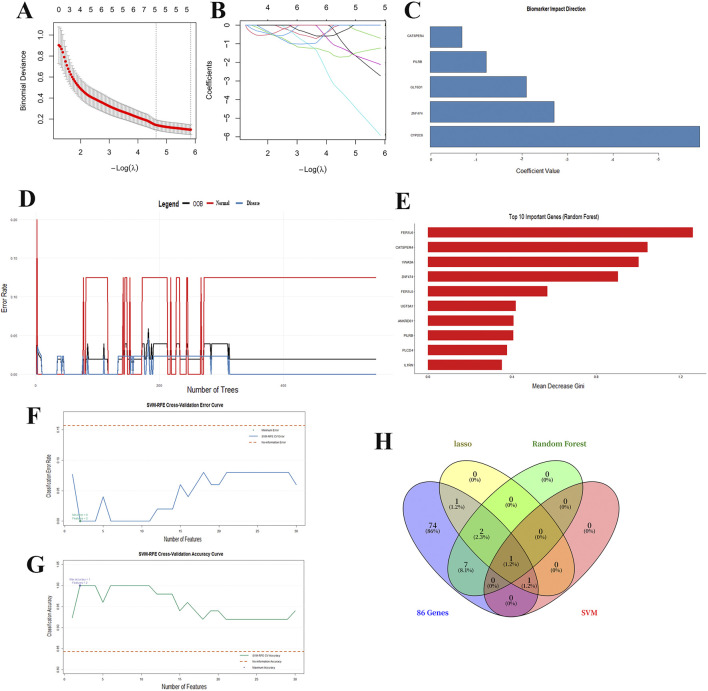
Identification of key diagnostic genes using integrated machine learning algorithms. **(A)** Cross-validation curve of the LASSO regression model for selecting the optimal penalty parameter (λ). **(B)** Coefficient profiles of candidate genes in the LASSO model. **(C)** Coefficient values of selected biomarkers in the LASSO model. **(D)** Error rate of the random forest model with increasing number of trees. **(E)** Top 10 important genes ranked by mean decrease Gini in the random forest model. **(F)** Cross-validation error curve of the SVM-RFE algorithm **(G)** Cross-validation accuracy curve of the SVM-RFE algorithm. **(H)** Venn diagram showing the intersection of candidate genes identified by LASSO, random forest, and SVM-RFE algorithms.

LASSO regression was first performed on the candidate gene expression matrix. The optimal penalty parameter (λ) was determined using tenfold cross-validation (Figure 5A), and the corresponding coefficient profiles and distribution plots were generated (Figures 5B,C). Subsequently, a random forest classification model was constructed. Model stability was evaluated by analyzing classification error rates across different numbers of decision trees (Figure 5D), which gradually plateaued as the number of trees increased. Gene importance was ranked based on the mean decrease in Gini index, and the top 10 genes contributing most to model performance were identified (Figure 5E).

In parallel, SVM-RFE was conducted to further refine feature selection. Cross-validation was used to assess classification error rates and accuracy under varying feature subset sizes (Figures 5F,G), allowing determination of the optimal number of features.

Finally, intersection analysis of genes identified by LASSO, random forest, and SVM-RFE revealed 12 shared feature genes, namely, ANKRD61, CATSPER4, CYP2C8, FER1L5, FER1L6, GLT6D1, IL1RN, PILRB, PLCD4, UGT3A1, VWA3A, and ZNF474 (Figure 5H). Partial overlap among algorithms supported the robustness of the selection strategy. These 12 genes were considered core candidate biomarkers for oligoasthenozoospermia and were subsequently used for model construction and biological validation.

### Predictive performance and clinical utility evaluation of machine learning models

Based on the selected feature genes, logistic regression, LASSO regression, random forest, and SVM models were constructed. Because no sufficiently appropriate external cohort was available, model performance was assessed by internal validation in the testing cohort.

**FIGURE 6:**
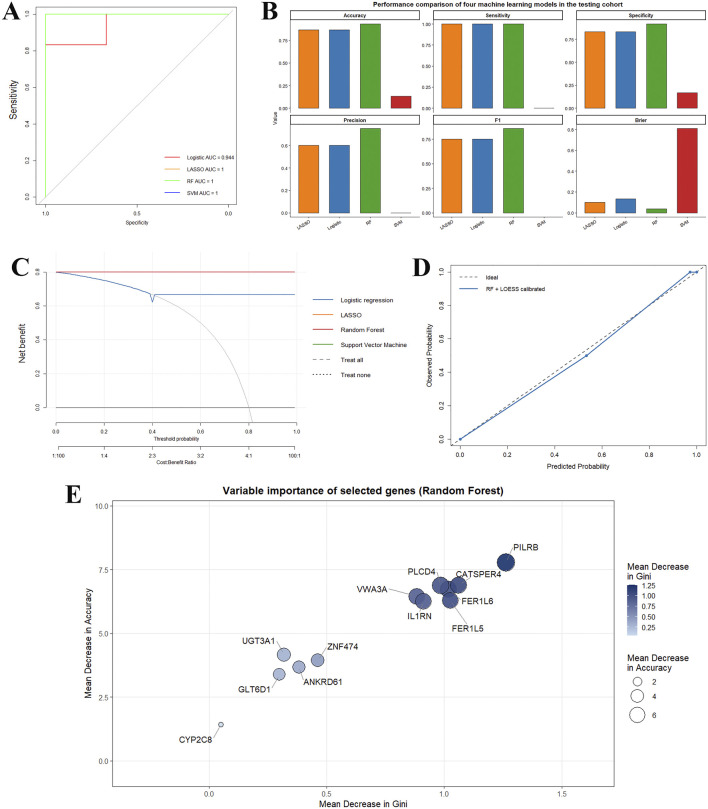
Performance evaluation and clinical utility assessment of machine learning–based diagnostic models. **(A)** Receiver operating characteristic (ROC) curves of logistic regression, LASSO, random forest, and support vector machine models in the testing cohort. **(B)** Comparison of accuracy, sensitivity, specificity, precision, F1 score, and Brier score among four machine learning models. **(C)** Decision curve analysis (DCA) demonstrating the net clinical benefit of different models across a range of threshold probabilities. **(D)** Calibration curve of the random forest model with LOESS smoothing, showing agreement between predicted and observed probabilities. **(E)** Variable importance plot of selected genes in the random forest model based on mean decrease in Gini and mean decrease in accuracy.

Receiver operating characteristic (ROC) curve analysis demonstrated strong internal discriminatory performance across all four models. The LASSO, random forest, and SVM models achieved an area under the curve (AUC) of 1.000, whereas the logistic regression model yielded an AUC of 0.944 (Figure 6A). Given the limited sample size and internal validation design, the near-perfect AUC values observed in several models may reflect optimistic performance estimation and potential overfitting rather than equivalent performance in independent cohorts. Therefore, these results should be interpreted cautiously.

Further comparison of performance metrics in the testing set, including accuracy, sensitivity, specificity, precision, F1 score, and Brier score, showed that the random forest model performed best across most evaluation indicators within the current internal validation setting (Figure 6B).

Decision curve analysis (DCA) was conducted to evaluate the potential clinical utility of each model. Across a broad range of threshold probabilities, the random forest and LASSO models exhibited higher net benefit compared with both “treat-all” and “treat-none” strategies (Figure 6C), suggesting favorable clinical applicability for risk stratification.

Calibration analysis of the random forest model showed acceptable agreement between predicted and observed probabilities, with the calibration curve closely approximating the ideal reference line (Figure 6D), suggesting reasonable performance within the current internal validation setting.

Variable importance analysis based on the random forest model identified PILRB, CATSPER4, FER1L6, PLCD4, and VWA3A as top-ranking contributors (Figure 6E), suggesting that these genes play critical roles in risk prediction for oligoasthenozoospermia.

### Differential expression of key genes and their association with immune-related transcriptomic signatures

**FIGURE 7:**
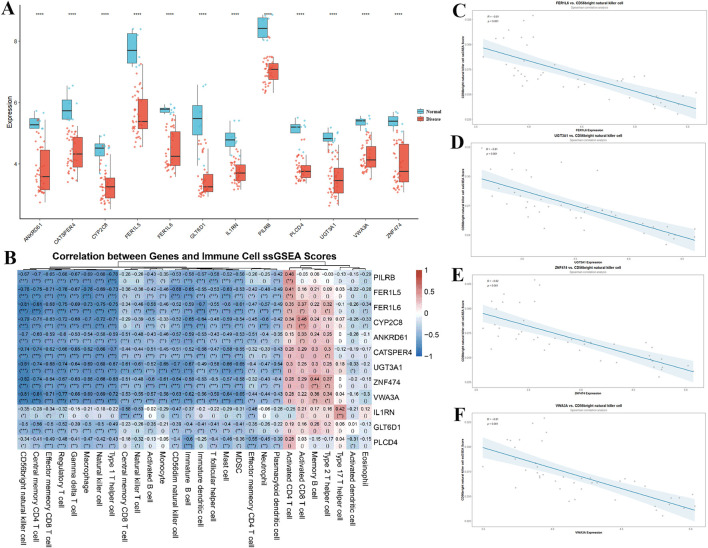
Expression patterns of hub genes and their associations with immune-related transcriptomic signatures in oligoasthenozoospermia. **(A)** Differential expression of 12 hub genes between normal and disease groups. Data are presented as boxplots with individual sample points. **(B)** Heatmap showing Spearman correlations between hub gene expression and immune-related ssGSEA signatures. **(C–F)** Representative scatter plots illustrating the negative correlations between selected hub genes (FER1L6, UGT3A1, ZNF474, and VWA3A) and the CD56bright natural killer cell–related signature. Correlation coefficients (R) and P values are indicated.

To further validate the expression characteristics and immunological relevance of the identified feature genes in oligoasthenozoospermia, their expression levels were compared between the disease and control groups. All 12 key genes showed significant differential expression between the two groups (Figure 7A). Compared with controls, these genes were consistently downregulated in the disease group (*P* < 0.05), suggesting their potential involvement in disease pathogenesis.

We next explored the relationship between key gene expression and immune-related transcriptomic signatures. Using ssGSEA-derived immune-related signature scores from the disease cohort, Spearman correlation analysis was performed to construct a gene–immune signature correlation heatmap (Figure 7B). The results demonstrated significant associations between multiple key genes and various immune-related signatures. Notably, several genes exhibited strong negative correlations with signatures related to natural killer (NK) cells, T Cell subsets, and dendritic cells.

To further evaluate representative gene–immune signature pairs, scatter plot analyses were conducted. FER1L6, UGT3A1, ZNF474, and VWA3A expression levels were all significantly negatively correlated with the CD56^bright^ NK cell–related signature (Figures 7C–F, *P* < 0.001). These findings suggest that the identified key genes may be associated with transcriptome-level immune microenvironment alterations in oligoasthenozoospermia.

### Functional annotation and integration of genetic regulatory evidence for candidate key genes

To systematically evaluate the potential biological functions of the identified key genes and their relevance to oligoasthenozoospermia, molecular functions, biological processes, and genetic regulatory evidence were comprehensively summarized (Table 1). The 12 key genes exhibited marked functional diversity.

**TABLE 1:** Functional annotation and integrated genetic regulatory evidence of hub genes associated with oligoasthenozoospermia.

Gene	Full name	Primary molecular function	Key biological process	Genetic evidence (QTL/disease)	Proposed role in spermatogenesis
ANKRD61	Ankyrin repeat domain 61	Protein–protein interaction scaffold	Cellular regulation and stress response	eQTLs in skin and nerve tissues	Upstream regulatory protein potentially influencing cellular homeostasis in reproductive tissues
ZNF474	Zinc finger protein 474	Transcriptional regulator	Gene expression control, neuronal development	Disease associations with neuromuscular disorders; eQTLs in smooth muscle–related tissues	Upstream transcriptional regulator affecting downstream reproductive pathways
FER1L6	Fer-1 like family member 6	Membrane trafficking and recycling	Vesicle transport, membrane dynamics	Testis exon-level eQTL; fibroblast eQTL	Regulation of membrane remodeling during germ cell development
FER1L5	Fer-1 like family member 5	Membrane fusion mediator	Myoblast fusion, endocytic recycling	Functional annotation evidence	Contribution to membrane fusion and structural integrity of developing sperm cells
CATSPER4	Cation channel sperm associated 4	Voltage-gated Ca²⁺ channel subunit	Calcium signaling, sperm motility	Strong disease association with spermatogenic failure; testis eQTL	Core regulator of sperm calcium influx essential for motility and fertilization
UGT3A1	UDP glycosyltransferase family 3 member A1	Steroid hormone conjugation	Phase II metabolism, hormone inactivation	Testis transcript-level eQTL	Regulation of local steroid hormone homeostasis in the testicular microenvironment
CYP2C8	Cytochrome P450 family 2 subfamily C member 8	Steroid and lipid metabolism	Fatty acid and hormone metabolism	Muscle eQTL; liver sQTL	Indirect modulation of sperm membrane composition and endocrine balance
VWA3A	von Willebrand factor A domain containing 3A	Cell adhesion–related scaffold protein	Cell–cell interaction, tissue architecture	Brain and pituitary eQTL/sQTL	Structural support within the reproductive axis and endocrine regulation
GLT6D1	Glycosyltransferase 6 domain containing 1	Protein glycosylation	Post-translational modification	Strong testis-specific eQTL and sQTL	Maintenance of sperm membrane protein stability and function
PLCD4	Phospholipase C delta 4	Phosphoinositide hydrolysis	Ca²⁺-dependent signal transduction	Brain exon eQTL; muscle tuQTL	Amplification of calcium signaling downstream of sperm ion channels
PILRB	Paired immunoglobulin-like type 2 receptor beta	Immune inhibitory receptor	Immune regulation and tolerance	Strong pQTL evidence (UK Biobank)	Regulation of immune microenvironment in the male reproductive tract
IL1RN	Interleukin 1 receptor antagonist	Anti-inflammatory cytokine	Inflammation suppression	Robust plasma pQTL evidence	Protection of spermatogenic niche from excessive inflammatory damage

ANKRD61 and ZNF474 are primarily involved in protein–protein interactions and transcriptional regulation, suggesting roles in gene expression control and cellular homeostasis. FER1L6 and FER1L5 participate in membrane trafficking and membrane fusion processes, which are closely related to cellular remodeling during spermatogenesis. CATSPER4, a subunit of the sperm-specific calcium channel complex, plays a central role in calcium influx regulation, sperm motility, and fertilization. UGT3A1 and CYP2C8 are involved in steroid hormone and lipid metabolism, potentially influencing endocrine balance within the testicular microenvironment. VWA3A and GLT6D1 are associated with cell adhesion and protein glycosylation, contributing to sperm membrane integrity and structural stability. PLCD4 participates in calcium-dependent signal transduction and may amplify intracellular signaling cascades in sperm cells. PILRB and IL1RN function in immune regulation and inflammatory suppression, highlighting the potential contribution of immune microenvironmental alterations to disease progression.

Integration of genetic regulatory evidence revealed that these genes exhibit stable eQTL, sQTL, or pQTL signals across multiple tissues, with several showing associations with reproductive phenotypes or related disorders. Collectively, the combined functional annotation and genetic regulatory data suggest that these key genes may contribute to oligoasthenozoospermia through coordinated regulation of calcium signaling, membrane remodeling, hormonal metabolic balance, and immune microenvironment homeostasis.

### Phenotypic and histopathological characterization of the oligoasthenozoospermia mouse model

**FIGURE 8:**
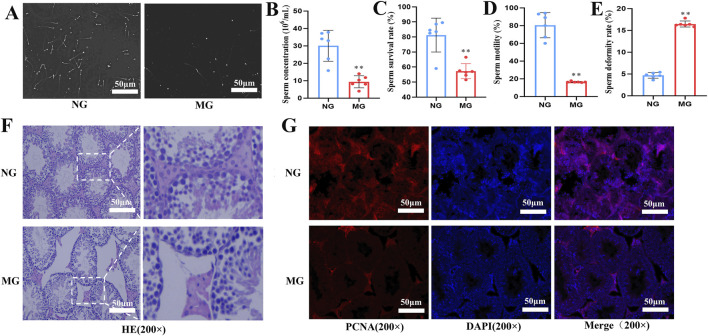
Phenotypic and histological validation of oligoasthenozoospermia mouse model **(A)** Representative microscopic images of epididymal sperm from normal group (NG) and model group (MG). Scale bar = 50 μm. **(B)** Sperm concentration. **(C)** Sperm survival rate. **(D)** Sperm motility. **(E)** Sperm deformity rate. **(F)** Representative hematoxylin and eosin (HE) staining of testicular sections in NG and MG (200×). Dashed boxes indicate magnified regions. Scale bar = 50 μm. **(G)** Immunofluorescence staining of proliferating cell nuclear antigen (PCNA, red) and DAPI (blue) in testicular tissues from NG and MG (200×). Merged images are shown on the right. Scale bar = 50 μm. Data are presented as mean ± SD. **P < 0.01 vs. NG.

To validate the effectiveness of model establishment, sperm quality parameters and testicular histological features were systematically evaluated in the normal group (NG) and model group (MG). Microscopic observation demonstrated that, compared with the NG, the MG exhibited a marked reduction in sperm count and impaired morphological integrity (Figure 8A).

Quantitative analysis further confirmed that sperm concentration, viability, and motility were significantly decreased in the MG, whereas the sperm abnormality rate was significantly increased (Figures 8B–E, *P* < 0.01), indicating that the model successfully reproduced the characteristic phenotype of oligoasthenozoospermia.

H&E staining revealed that seminiferous tubules in the NG displayed intact architecture, tightly arranged spermatogenic epithelium, and orderly distribution of germ cells at different developmental stages. In contrast, the MG showed disorganized seminiferous tubules, dilated lumens, reduced numbers of spermatogenic cells, and focal cell detachment with vacuolar degeneration (Figure 8F), suggesting significant impairment of spermatogenic function.

Immunofluorescence staining for the proliferation marker proliferating cell nuclear antigen (PCNA) further demonstrated that PCNA-positive signals were widely distributed in the basal and intermediate layers of seminiferous epithelium in the NG. However, PCNA expression was markedly reduced in the MG, with a significant decrease in the number of positive cells (Figure 8G), indicating diminished proliferative activity of spermatogenic cells.

Collectively, these findings demonstrate that chronic smoke exposure combined with ethanol intervention successfully induced a stable oligoasthenozoospermia phenotype characterized by reduced sperm count, impaired motility, structural damage to seminiferous tubules, and decreased germ cell proliferation. This model provides a reliable *in vivo* platform for biological validation of transcriptomic findings and for future mechanistic investigations and therapeutic evaluation.

#### Transcriptomic profiling and molecular validation of key candidate genes

**FIGURE 9:**
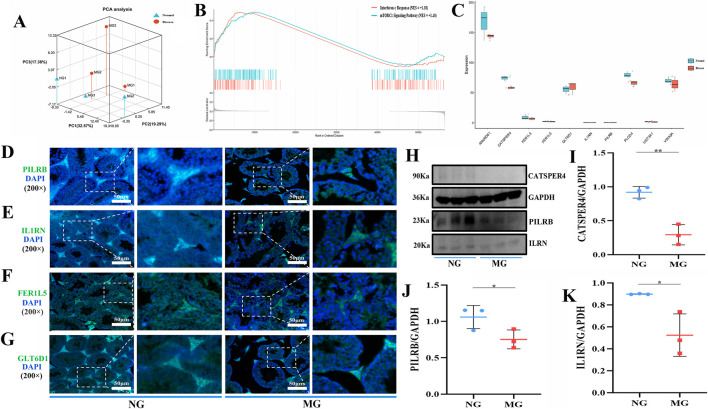
Multi-level molecular validation of key pathways and hub genes in the oligoasthenozoospermia mouse model **(A)** Three-dimensional principal component analysis (PCA) of testicular transcriptome profiles from normal group (NG) and model group (MG). **(B)** Gene set enrichment analysis (GSEA) plots showing enrichment of interferon-γ response and mTORC1 signaling pathways. **(C)** Differential expression of representative hub genes between NG and MG groups. **(D–G)** Immunofluorescence staining of PILRB, IL1RN, FER1L5, and GLT6D1 (green) in testicular tissues from NG and MG. Nuclei were counterstained with DAPI (blue). Scale bar = 50 μm. **(H)** Western blot analysis of CATSPER4, PILRB, and IL1RN protein expression in NG and MG groups. GAPDH was used as a loading control. **(I–K)** Quantitative analysis of CATSPER4, PILRB, and IL1RN protein expression normalized to GAPDH. Data are presented as mean ± SD. *P < 0.05, **P < 0.01 vs. NG.

To further characterize global transcriptomic alterations between groups, principal component analysis (PCA) was performed on testicular RNA-sequencing data. The results demonstrated a clear separation between the normal group (NG) and model group (MG) in three-dimensional space (Figure 9A), indicating substantial differences in overall gene expression profiles.

Gene set enrichment analysis (GSEA) revealed that interferon-γ and mTORC1 signaling pathways were significantly enriched in the MG (Figure 9B), suggesting enhanced immune-inflammatory activation and aberrant metabolic signaling in the oligoasthenozoospermia model. These findings were consistent with the bioinformatics results derived from human transcriptomic datasets.

Expression analysis of key candidate genes within the RNA-seq dataset showed that ANKRD61, CATSPER4, FER1L6, PLCD4, UGT3A1, and VWA3A were downregulated in the MG compared with the NG (Figure 9C), in agreement with the integrated bioinformatics analysis.

To validate protein-level alterations in testicular tissue, immunofluorescence staining was performed for PILRB, IL1RN, FER1L5, and GLT6D1. In the NG, these proteins exhibited strong positive signals, primarily localized within interstitial cells and spermatogenic cell regions. In contrast, fluorescence intensity was markedly reduced in the MG (Figures 9D–G), indicating decreased protein expression.

Western blot analysis further confirmed that CATSPER4, PILRB, and IL1RN protein levels were significantly lower in the MG compared with the NG (Figures 9H–K,P < 0.05), consistent with immunofluorescence findings.

Collectively, transcriptomic profiling and molecular validation consistently demonstrated that the oligoasthenozoospermia mouse model exhibits significant alterations in gene expression patterns, pathway activity, and key functional protein levels. These findings further support the biological relevance of the identified candidate genes in oligoasthenozoospermia, although they do not constitute comprehensive mechanistic validation.

## Discussion

### Major findings and innovative contributions

In this study, we integrated multi-cohort transcriptomic analysis, bioinformatics screening, machine learning modeling, and *in vivo* experimental validation to systematically characterize the molecular landscape of oligoasthenozoospermia and to construct a coordinated pathological framework centered on immune–inflammatory activation, metabolic reprogramming, and impaired spermatogenesis.

Based on the integrated analysis of the GSE45887 and GSE45885 datasets, we identified a highly consistent differential expression signature following batch correction and normalization. Weighted gene co-expression network analysis (WGCNA) further revealed key gene modules significantly associated with disease phenotype, providing a robust foundation for candidate gene prioritization. Functional enrichment analyses demonstrated that immune- and metabolism-related pathways—including complement signaling pathway, IL6–JAK–STAT3 signaling pathway, interferon-γ signaling pathway, and mTORC1/PI3K–AKT–mTOR signaling pathway exhibited an overall upward trend in the disease group, whereas spermatogenesis-related programs were markedly suppressed. These findings suggest that disruption of immune homeostasis and metabolic signaling may cooperatively contribute to the pathogenesis of oligoasthenozoospermia.

Using an integrative feature selection strategy combining LASSO regression, random forest, and SVM-RFE, we identified 12 key candidate genes and constructed predictive models with strong apparent discriminatory performance. In 7:3 internal validation, the models showed favorable performance within the current dataset, with random forest exhibiting the best overall results and clinical net benefit. However, given the limited overall sample size, the small number of control samples, and the lack of external validation, these findings should be interpreted cautiously.

Importantly, we established a smoke- and ethanol-induced mouse model of oligoasthenozoospermia to evaluate the biological relevance of the computational findings at phenotypic, histological, transcriptomic, and protein levels. The model reproduced reduced sperm count and motility, structural testicular damage, and downregulation of key candidate genes, consistent with the human transcriptomic results. This integrative design established a multi-level evidence framework linking population-level data mining with biological support *in vivo*, rather than exhaustive mechanistic validation.

Collectively, our findings reveal a coordinated imbalance characterized by immune-inflammatory activation, metabolic pathway dysregulation, and suppression of spermatogenic programs. This systems-level framework highlights the potential translational value of our study in elucidating the molecular basis of oligoasthenozoospermia and identifying interpretable candidate biomarkers.

### A coordinated molecular framework linking immune–inflammatory activation, metabolic signaling dysregulation, and impaired spermatogenesis

Our findings suggest that oligoasthenozoospermia is unlikely to be driven by a single molecular abnormality. Instead, it appears to arise from a coordinated imbalance involving enhanced immune–inflammatory responses, dysregulated metabolic and growth signaling, and suppression of spermatogenic programs, collectively forming a self-amplifying pathological network (Collodel et al., 2025; Batra et al., 2021). Based on multi-dimensional transcriptomic analyses, we propose a mechanistic framework in which immune activation, metabolic reprogramming, and spermatogenic dysfunction are serially connected and mutually reinforcing.

At the immune–inflammatory level, GSVA and GSEA consistently demonstrated persistent activation of complement signaling, IL6–JAK–STAT3 signaling, and interferon response pathways in the disease group. In parallel, ssGSEA suggested substantial variation in immune-related transcriptomic signatures, which may reflect potential alteration of the testicular immune microenvironment. However, given that these inferences were derived from bulk tissue transcriptomic data, they should be interpreted cautiously and not as direct evidence of immune cell composition changes. Sustained inflammatory signaling may compromise the integrity of the blood–testis barrier and disrupt communication between Sertoli cells and germ cells, thereby inducing structural and functional remodeling of the reproductive niche (Li et al., 2025). This immune disequilibrium likely establishes the pathological foundation for subsequent metabolic disturbances and impaired spermatogenesis.

At the level of metabolic and growth signaling, we observed significant activation of the mTORC1 and PI3K–AKT–mTOR pathways in the disease group. As a central regulator of cellular energy metabolism, protein synthesis, and autophagy, aberrant mTOR activation often reflects compensatory stress responses or metabolic imbalance (Silva et al., 2019; Corda et al., 2025; Yao et al., 2025; Simonik et al., 2025). Under chronic inflammatory stimulation, immune mediators may continuously stimulate mTOR-related pathways, altering energy allocation and homeostatic regulation in Sertoli cells and developing germ cells (Doersch et al., 2021; Rhouma et al., 2024). Although such metabolic reprogramming may initially represent an adaptive response, prolonged dysregulation may ultimately lead to cellular exhaustion and destabilization of the spermatogenic microenvironment.

At the level of spermatogenic function, gene sets associated with spermatogenesis were significantly downregulated in patients. Consistently, the mouse model exhibited reduced PCNA expression and histological evidence of seminiferous tubule disorganization and germ cell loss. These findings suggest that immune and metabolic abnormalities may converge to suppress germ cell proliferation, impair differentiation processes, and disrupt structural integrity of the seminiferous epithelium, thereby leading to systemic spermatogenic dysfunction (Roustaee et al., 2024). Immune-mediated microenvironmental damage combined with metabolic instability may jointly compromise spermatogonial stem cell maintenance and sperm maturation efficiency (Rokade and Madan, 2016), ultimately manifesting as reduced sperm count, decreased motility, and increased morphological abnormalities.

Taken together, we propose that oligoasthenozoospermia may evolve through a cascade initiated by sustained immune activation, amplified by metabolic and growth signaling dysregulation, and culminating in progressive spermatogenic failure. Rather than acting independently, immune imbalance, metabolic disturbance, and impaired spermatogenesis likely form a dynamic feedback network that drives disease progression. This integrated mechanistic framework not only advances our understanding of the molecular pathology of oligoasthenozoospermia but also provides a conceptual basis for future therapeutic strategies targeting the immune–metabolic–spermatogenic axis.

### Functional modular interpretation of key candidate genes

Integrating bioinformatic screening, *in vivo* validation, and previous literature evidence, the key genes identified in this study exhibit clear functional modularity. These modules primarily involve calcium signaling, membrane remodeling and vesicle trafficking, metabolic microenvironment regulation, and immune–inflammatory modulation. Importantly, these biological processes are not independent; rather, they act cooperatively to maintain spermatogenic homeostasis. Their coordinated disruption may collectively drive the development of oligoasthenozoospermia.

### Calcium signaling dysfunction and impaired sperm motility and fertilization (CATSPER4, PLCD4)

Calcium signaling is essential for sperm capacitation, hyperactivation, and fertilization. The CATSPER channel complex represents the principal sperm-specific Ca^2+^ channel, playing a decisive role in flagellar motility regulation (Nicolli et al., 2026). Previous studies have demonstrated that CATSPER4 dysfunction markedly impairs calcium influx, resulting in reduced sperm motility and fertilization capacity (Jin et al., 2026; Hwang et al., 2026). In the present study, CATSPER4 was consistently downregulated in both human transcriptomic datasets and the mouse model, and protein-level reduction was confirmed by Western blot. Correspondingly, model mice exhibited significantly decreased sperm motility, aligning with reduced CATSPER4 expression.

PLCD4, a member of the phospholipase C family, mediates phosphatidylinositol hydrolysis and participates in calcium signal amplification (Marcos, 2024; Batra et al., 2020). Its downregulation may further weaken intracellular calcium signaling efficiency in sperm cells. Together with the observed suppression of spermatogenesis-related gene sets, these findings suggest that coordinated dysregulation of CATSPER4 and PLCD4 may disrupt the sperm calcium signaling network, leading to impaired motility and fertilization potential—representing a critical molecular basis of oligoasthenozoospermia.

### Abnormal membrane remodeling and vesicle trafficking (FER1L5, FER1L6)

Spermatogenesis is characterized by extensive cellular morphological remodeling and membrane restructuring, processes that depend on membrane fusion and structural stability during germ cell differentiation (Nir et al., 2023; Grinshtain et al., 2022).

In this study, FER1L5 and FER1L6 were consistently downregulated, and reduced FER1L5 protein expression was validated by immunofluorescence. Histological analysis revealed disorganized seminiferous tubules and loosened germ cell arrangement in model mice, consistent with impaired membrane remodeling. Previous studies have reported that FER1L family members contribute to membrane stability and developmental stage transitions during cell differentiation (Nir et al., 2023; Grinshtain et al., 2022). Therefore, reduced FER1L5/FER1L6 expression may compromise germ cell membrane integrity and vesicle transport efficiency, thereby interfering with sperm morphogenesis and maturation.

### Disruption of hormonal and metabolic microenvironment (UGT3A1)

UGT3A1, a member of the UDP-glycosyltransferase family, participates in the conjugation and inactivation of steroid hormones and endogenous metabolites, thereby contributing to testicular hormonal balance.

In both human datasets and the mouse model, UGT3A1 expression was significantly downregulated, suggesting impaired local hormone metabolism. Hormonal imbalance has been reported to disrupt Sertoli cell function and destabilize the spermatogenic niche (Shah et al., 2021). Moreover, altered steroid metabolism may indirectly affect sperm membrane composition and signaling capacity. Given the reduced sperm count and increased morphological abnormalities observed in model mice, decreased UGT3A1 expression may contribute to spermatogenic dysfunction through disruption of endocrine and metabolic homeostasis.

### Impaired immune suppression and inflammatory regulation (PILRB, IL1RN)

As an immune-privileged organ, the testis relies on tightly regulated immunosuppressive and anti-inflammatory mechanisms to preserve germ cell integrity. PILRB functions as an inhibitory immune receptor, while IL1RN acts as an interleukin-1 receptor antagonist; both are involved in negative regulation of inflammatory responses (Pillai and Fang, 2025; Wang et al., 2024).

In this study, PILRB and IL1RN were significantly downregulated in the disease group, and reduced protein expression was confirmed by immunofluorescence and Western blot. Immune signature analysis further suggested altered immune-related transcriptomic patterns, with significant associations between key genes and signatures related to NK cells and T Cells. These findings suggest that decreased expression of PILRB and IL1RN may weaken local immune tolerance within the testis, impair termination of inflammatory signaling, and exacerbate immune-mediated spermatogenic damage. Consistent with GSEA results showing activation of immune–inflammatory pathways, immune dysregulation likely represents a fundamental immunological basis of oligoasthenozoospermia.

### Biological significance of gene–immune signature associations

In addition to identifying key molecular signatures, this study systematically characterized the immune microenvironment of oligoasthenozoospermia using ssGSEA and further explored the correlations between candidate genes and immune-related transcriptomic signatures. The results revealed coordinated alterations across multiple immune-related signatures, indicating a substantial reshaping of the testicular immune transcriptomic landscape in the disease state. Importantly, most key genes exhibited significant correlations with immune-related signature scores, highlighting a close coupling between transcriptional dysregulation and local immune imbalance.

Correlation heatmap analysis demonstrated that PILRB, FER1L5, FER1L6, CATSPER4, and UGT3A1 were negatively correlated with several adaptive immune-related signatures, including T Cell populations, natural killer (NK) cells, and dendritic cells. In contrast, associations with certain innate immune-related signatures were weaker or directionally heterogeneous. This broad correlation pattern suggests that the downregulation of reproductive-associated genes in oligoasthenozoospermia is accompanied by systemic immune microenvironment remodeling rather than isolated changes in a single immune cell subset.

Notably, scatter plot analysis further showed significant negative correlations between multiple key genes and the CD56^bright^ NK cell–related signature. Because this analysis was based on bulk transcriptomic inference, these results should be interpreted as associations with immune-related transcriptional patterns rather than direct evidence of increased NK cell abundance. Nevertheless, they raise the possibility that innate immune-related activity may be linked to dysregulation of the local testicular microenvironment.

Moreover, PILRB and IL1RN, both implicated in immune suppression and inflammatory regulation, were significantly downregulated in the disease group and strongly associated with immune-related transcriptomic signature patterns. PILRB functions as an inhibitory immune receptor involved in maintaining immune tolerance (Hees et al., 2026), whereas IL1RN antagonizes IL-1 signaling to restrain inflammatory cascades (Pillai and Fang, 2025; Yang et al., 2018). Reduced expression of these molecules may weaken local immunoregulatory capacity within the testis, facilitating persistent low-grade inflammation that ultimately compromises spermatogenic integrity.

Collectively, these findings indicate that oligoasthenozoospermia may not solely reflect intrinsic germ cell defects but also involve sustained immune microenvironment dysregulation. The coordinated disruption of key reproductive genes and immune-related transcriptomic signatures suggests a “gene–immune microenvironment” interaction axis, wherein transcriptional alterations may be associated with immune-related signaling activity and inflammatory regulation, thereby indirectly affecting testicular homeostasis and spermatogenesis. This integrated perspective provides mechanistic insight into the immunological contribution to male infertility and supports the potential development of therapeutic strategies targeting the immune–reproductive axis.

### Model robustness and translational applicability

Based on integrative multi-cohort transcriptomic analysis and systematically selected feature genes, this study established several classification models, including logistic regression, LASSO regression, random forest, and support vector machine, and performed internal validation in a held-out test set. Except for the logistic regression model, the remaining models showed strong apparent discriminatory capacity, with the random forest model achieving the best overall performance in terms of accuracy, sensitivity, specificity, and composite evaluation metrics within the current dataset. However, because no sufficiently appropriate external human validation cohort was available, these results should be interpreted as preliminary.

ROC curve analysis further showed that the LASSO, random forest, and support vector machine models achieved high AUC values in internal validation, underscoring the apparent discriminative potential of the selected gene signature within the current dataset. However, given the limited sample size and the near-perfect performance observed in some models, these results may reflect optimistic performance estimation, and the possibility of overfitting cannot be fully excluded. Compared with single-biomarker strategies, multi-gene modeling may reduce the impact of inter-individual biological heterogeneity and may improve model adaptability in complex clinical contexts, but these advantages still require confirmation in larger independent cohorts.

From a clinical utility perspective, decision curve analysis demonstrated that both the random forest and LASSO models provided consistent net benefit across a broad range of threshold probabilities, outperforming conventional “treat-all” or “treat-none” strategies. Moreover, the calibration curve of the random forest model closely approximated the ideal reference line, indicating good agreement between predicted probabilities and observed outcomes. Together, these findings suggest potential utility of the model within the current analytical setting, although external validation will be required before clinical application.

Importantly, the predictive model was constructed based on biologically grounded core genes, several of which were examined at the transcriptomic and protein levels in the animal model. This multi-layered validation strategy—linking population-level data, *in vivo* modeling, and molecular experimentation—enhances the biological plausibility of the model; however, the animal experiments in the present study were designed to provide biological support for selected transcriptomic findings rather than comprehensive mechanistic confirmation.

From a translational medicine perspective, the proposed multi-gene predictive framework holds considerable application potential. The model is compatible with high-throughput transcriptomic platforms and may serve as a foundation for the development of standardized molecular testing panels. Furthermore, the core genes are enriched in pathways related to immune regulation, metabolic homeostasis, and spermatogenesis, providing mechanistic targets for future stratified intervention strategies. With the advancement of liquid biopsy technologies and multi-omics diagnostic platforms, this integrative model may be further optimized for minimally invasive or non-invasive clinical implementation.

It should also be noted that the candidate biomarkers identified in the present study were derived from testicular tissue transcriptomic data, which are biologically informative for local spermatogenic dysfunction but are not directly suitable for routine clinical screening because testicular tissue acquisition is invasive. Therefore, the current findings should be interpreted as a tissue-level candidate biomarker framework rather than an immediately deployable diagnostic assay. Future translational studies should investigate whether these candidate genes, their encoded proteins, or related downstream molecular signatures can be reproducibly detected in more clinically accessible biospecimens, such as semen, seminal plasma, blood, or extracellular vesicles. Validation in such minimally invasive sample types will be essential for assessing the true clinical applicability of the proposed biomarker panel.

## Limitations and future perspectives

Despite the integrative design combining multi-cohort transcriptomic analysis, machine learning modeling, and *in vivo* validation, several limitations should be acknowledged. First, the human transcriptomic analysis was based on a limited number of publicly available datasets, and the completeness and homogeneity of clinical annotations in these datasets were constrained by the original study designs. In particular, the number of normal control samples available in the public testicular transcriptomic datasets was small, which may have reduced statistical power and increased the instability of performance estimation. Because publicly available human testicular transcriptomic datasets specifically focused on oligoasthenozoospermia are extremely scarce, potential phenotypic heterogeneity cannot be fully excluded. Second, although several key genes were examined at the transcriptional and protein levels in the mouse model, the *in vivo* validation was not designed to comprehensively assess all mechanistic layers. In particular, measurements of reproductive hormones, oxidative stress markers, and blood–testis barrier integrity were not included in the present study. Therefore, the animal experiments should be interpreted as providing biological support for the relevance of selected candidate genes and pathways rather than exhaustive mechanistic validation. Third, although the proposed multi-gene models showed strong internal discriminatory performance, no sufficiently appropriate independent external human cohort was available for validation. The scarcity of publicly available testicular transcriptomic datasets, together with limited sample size and heterogeneous clinical annotations, restricted the feasibility of external validation in the present study. Therefore, the current model results should be interpreted as preliminary evidence, and the possibility of overfitting cannot be fully excluded, particularly in light of the near-perfect AUC values observed in some models. In addition, the immune-related findings were inferred from bulk testicular transcriptomic data using ssGSEA-based signature analysis rather than direct cellular measurements. Accordingly, these results do not constitute definitive evidence of immune cell composition changes, and future studies should incorporate marker-based validation, immunohistochemistry, flow cytometry, or single-cell transcriptomic approaches to confirm the observed immune-related patterns. Fourth, while the smoke- and ethanol-induced mouse model successfully recapitulated spermatogenic dysfunction, it may not fully capture the etiological heterogeneity and multifactorial complexity of human oligoasthenozoospermia. Therefore, the present findings should be interpreted as exploratory transcriptomic evidence derived from currently available public resources rather than as a clinically definitive molecular classification of oligoasthenozoospermia. In addition, the candidate biomarkers were identified from testicular tissue transcriptomes, which provide important biological insight into local pathological changes but are not directly convenient for routine clinical testing. Therefore, whether these tissue-derived candidates can be translated into semen-, seminal plasma-, blood-, or extracellular vesicle–based assays remains to be established in future studies.

Future studies should incorporate large-scale, multicenter prospective cohorts to externally validate the identified gene signature and predictive model. Multi-omics integration, including proteomics and metabolomics, will further refine molecular subtyping and mechanistic interpretation. Functional investigations using gene knockout or overexpression models are warranted to delineate the causal roles of key genes in regulating spermatogenesis and immune microenvironment remodeling. From a translational perspective, the development of minimally invasive diagnostic strategies based on blood- or semen-derived biomarkers, combined with intelligent analytical platforms, may facilitate the clinical implementation of the proposed immune–metabolic–spermatogenic axis framework and support precision management of oligoasthenozoospermia.

## Conclusion

This study identifies and preliminarily supports a candidate molecular biomarker signature for oligoasthenozoospermia through integrated public transcriptomic analysis combined with multi-level experimental validation. By systematically prioritizing candidate genes across the currently available datasets and examining their biological relevance *in vivo*, we identified a 12-gene candidate panel associated with the core molecular alterations underlying the disease phenotype.

Beyond statistical discrimination, the proposed signature shows biological coherence, consistency across analytical strategies, and translational plausibility. The integration of immune-related transcriptomic signature profiling with network-based gene selection strengthens the interpretability of the identified markers, while internal validation provides preliminary support for their diagnostic potential. Importantly, concordant transcriptional and protein-level changes observed in the animal model provide biological support for the relevance of this gene panel.

Rather than serving solely as predictive variables, these biomarkers reflect an underlying coordinated immune–metabolic–spermatogenic dysregulation pattern, thereby linking molecular stratification with pathophysiological context. This integrative framework enhances the credibility of the proposed biomarkers and lays the groundwork for future development of standardized molecular diagnostic assays in male infertility.

Further validation in larger, independent, and more clinically homogeneous cohorts, together with external evaluation of model performance, will be required to facilitate clinical translation. In addition, future studies should determine whether the tissue-derived candidate biomarkers identified here can be adapted to minimally invasive assays using semen-, seminal plasma-, blood-, or extracellular vesicle–based samples. Nevertheless, the present study provides a preliminary biomarker-driven framework for future molecular stratification and diagnostic development in oligoasthenozoospermia.

## Data Availability

The datasets presented in this study can be found in online repositories. The names of the repository/repositories and accession number(s) can be found below: PRJNA1439735 (Bioproject, NCBI).
